# Gastrointestinal Symptoms are Still Prevalent and Negatively Impact Health-Related Quality of Life: A Large Cross-Sectional Population Based Study in The Netherlands

**DOI:** 10.1371/journal.pone.0069876

**Published:** 2013-07-29

**Authors:** Merel M. Tielemans, Jeroen Jaspers Focks, Leo G. M. van Rossum, Ties Eikendal, Jan B. M. J. Jansen, Robert J. F. Laheij, Martijn G. H. van Oijen

**Affiliations:** 1 Department of Gastroenterology and Hepatology, Radboud University Medical Center, Nijmegen, the Netherlands; 2 Department of Gastroenterology and Hepatology, University Medical Center Utrecht, Utrecht, the Netherlands; 3 Department of Cardiology, Radboud University Medical Center, Nijmegen, the Netherlands; 4 Department of Epidemiology, Biostatistics and Health Technology Assessment, Radboud University Medical Center, Nijmegen, the Netherlands; 5 Department of Gastroenterology and Hepatology, Elkerliek Hospital, Helmond, the Netherlands; 6 Division of Digestive Diseases, David Geffen School of Medicine at UCLA, Los Angeles, California, United States of America; Iran University of Medical Sciences, Islamic Republic of Iran

## Abstract

**Background:**

Over the last decades important risk factors for gastrointestinal symptoms have shifted, which may have changed its population prevalence. The aim of this study was to assess the current prevalence of gastrointestinal symptoms, appraise associated factors and assess health-related quality of life in the general population.

**Methods:**

A total of 51,869 questionnaires were sent to a representative sample of the Dutch adult general population in December 2008. Demographic characteristics, gastrointestinal symptoms, health-related quality of life, medication use and co-morbidity were reported. We used multivariable logistic regression analysis to determine factors associated with gastrointestinal symptoms.

**Results:**

A total of 18,317 questionnaires were returned, and 16,758 were eligible for analysis. Prevalence of gastrointestinal symptoms was 26%. Most frequent symptoms were bloating (63%), borborygmi (60%) and flatulence (71%). Female gender (adjusted OR (aOR) 1.59, 95% CI 1.43–1.77), asthma/COPD (aOR 1.47, 95% CI 1.21–1.79), use of paracetamol (aOR 1.33, 95% CI 1.20–1.47), antidepressants (aOR 1.56, 95% CI 1.22–2.00) and acid-suppressive medication were independently associated with presence of gastrointestinal symptoms. Age over 65 years (aOR 0.75, 95% CI 0.65–0.87), and use of statins (aOR 0.75, 95% CI 0.61–0.93) were associated with a lower prevalence of gastrointestinal symptoms. Respondents with gastrointestinal symptoms had a lower mean health-related quality of life of 0.81 (SD = 0.21) compared to 0.92 (SD = 0.14) for persons without gastrointestinal symptoms (P<0.01).

**Conclusions:**

Prevalence of gastrointestinal symptoms in the Dutch community is high and associated with decreased health-related quality of life.

## Introduction

Gastrointestinal symptoms are highly prevalent in the general population and are a frequent cause for consultation of a general practitioner [1]. Individuals with gastrointestinal symptoms contribute heavily to healthcare utilization and budgeting. The yearly costs for individual patients with gastrointestinal symptoms are steep. For example, in the United States average direct healthcare costs for a number of symptoms such as constipation ($7522), functional abdominal pain ($7646) and irritable bowel syndrome ($5049) are considerable [2].

Large population studies in Western countries reported a widely ranging prevalence of dyspepsia from 10% to more than 50% [1], [3]–[12]. However, these studies were performed about 20 years ago and the risk factor profile for gastrointestinal symptoms has shifted since. For example, the incidence of *Helicobacter pylori* has rapidly decreased in the industrialized world [13], [14], while use of proton pump inhibitors (PPIs) has been on the rise [15]–[17]. Simultaneously, use of gastrotoxic medication, e.g. non-steroidal anti-inflammatory drugs (NSAIDs) and low-dose aspirin, is high [18]. Finally, there is a global epidemic of obesity, which is associated with gastrointestinal symptoms and disorders, especially gastroesophageal reflux disease (GERD) [19], [20]. The overall prevalence of upper gastrointestinal symptoms ranged from 24% to 45% in a recent study in 13 European countries [21]. Although performed in the current era, this study emphasized on socioeconomic factors, and did not report associations between gastrointestinal symptoms and modifiable factors such as BMI and smoking on an individual level [21].

Health-related quality of life is an important parameter in modern medicine and refers to the extent that an individual’s physical, emotional and social well-being is affected by a medical condition and its treatment. Individuals with gastrointestinal symptoms report a lower health-related quality of life [22]–[24], but this has been mainly studied in a subgroup of patients that have presented to a healthcare provider, which may not be a representative group. The exact impact of gastrointestinal symptoms on all domains of health related quality of life in the general –including non-healthcare visiting- population remains unclear.

Given abovementioned considerations, new data on the prevalence of gastrointestinal symptoms in the general population are warranted. We hypothesize that the prevalence, despite all changes, has remained stable. The aims of our study were to assess: 1) the prevalence of gastrointestinal symptoms in the general population; 2) factors associated with presence of gastrointestinal symptoms; and 3) the effect of gastrointestinal symptom presence on health-related quality of life.

## Materials and Methods

### Study Population

We sent 51,869 questionnaires by postal mail to a sample of the Dutch population in December 2008. Invited subjects were at least 18 years and randomly selected from municipal databases of five different municipalities. These villages and cities were selected on their geographical location in The Netherlands, in order to fetch a representative sample. We included returned questionnaires until the end of March 2009. We excluded returned questionnaires with (1) missing of all baseline variables, (2) missing of all gastrointestinal symptoms, (3) missing of the primary outcome measure, or (4) unreadable input about medication use.

The Medical Ethical Committee of the Radboud University Nijmegen assessed the proposal of this study and concluded that it could be waived for ethical review, as questionnaires were returned and stored anonymously, and (non-)responders would not be contacted again. For this reason, we did not obtain written informed consent of all participants.

### Questionnaire

The questionnaire we used was specifically designed to assess demographic information, gastrointestinal symptoms, medication use, healthcare visits, and health-related quality of life, and has been used before [15], [25], [26]. Respondents were asked whether they suffer from gastrointestinal symptoms in general and subsequently for presence of 26 gastrointestinal symptoms including nausea, early satiety, bloating, constipation and diarrhoea. Severity of gastrointestinal symptoms was assessed on a seven-point Likert scale (0 = absent, 1 = almost absent, 2 = mild, 3 = moderate, 4 = moderately severe, 5 = severe, 6 = very severe) during the preceding four weeks [27]. A symptom was considered to be present when scored ≥2.

Our primary outcome was the presence or absence of gastrointestinal symptoms, which was assessed with the question: “Do you experience gastrointestinal complaints?” and had to be answered with either “yes” or “no”. Secondary outcomes were type of gastrointestinal symptoms experienced in the preceding four weeks and health-related quality of life, which was assessed with the validated EQ-5D questionnaire [28]. The EQ-5D comprises 5 domains of health status: mobility, self-care, usual activities, pain/discomfort and anxiety/depression. Each domain has 3 degrees of severity: no problems, some problems or extreme problems. The EQ-5D tariffs were calculated by using Dutch coefficients for Time Trade Off tariffs [29]. The EQ-5D questionnaire also contains a visual analogue scale (EQ VAS), ranging from the worst imaginable health status to the best imaginable health status.

We defined excessive consumption of alcohol as ≥14 units (women) or ≥21 units (men) per week [30]. Body mass index (BMI in kg/m^2^) was categorized in <25 (normal weight) and 25 or more (overweight or obese) [31]. Excessive coffee consumption was defined as 42 cups or more per week [25], [32].

### Statistical Analysis

We analysed data using Statistical Package for the Social Sciences (SPSS), version 16.0 (IBM Corporation, New York, United States). Frequency tables were provided for respondents’ characteristics and for secondary outcomes. Pearson’s chi squared (χ^2^) analysis was used to compare categorical variables between respondents with and without presence of gastrointestinal symptoms. Continuous variables were compared between the two groups using Student’s T-test or Mann-Whitney U method whenever appropriate. We calculated presence of various symptoms at various ages by calculating symptom presence per 10 years. Univariable and multivariable logistic regression analysis were performed to identify factors associated with gastrointestinal symptoms. Odds ratios and 95% confidence intervals were stated. Covariates were included in multivariable regression analysis based on a predefined conceptual model that was based on published literature. We also included covariates if they were univariably associated with the primary outcome (p<0.01).

Health-related quality of life was compared between respondents with and without gastrointestinal symptoms. The 5 domains of the EQ-5D questionnaire were compared using chi squared (χ^2^) analysis. Dutch utility scores for every individual symptom in persons reporting gastrointestinal symptoms were calculated to assess the impact of an individual symptom on health-related quality of life. Correlation between gastrointestinal symptom score (VAS) and health-related quality of life (EQ VAS) was calculated with a Spearman correlation. A p-value<0.01 was assumed to be statistically significant.

## Results

**Figure 1:**
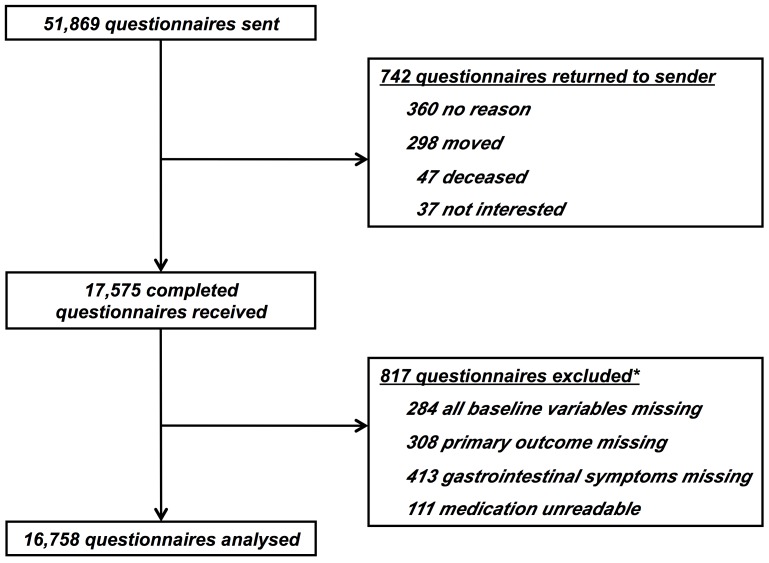
Flow chart. *Some respondents fulfilled more than 1 exclusion criterion.

A total of 18,317 (35%) questionnaires were returned, of which 742 returned unopened and uncompleted. After applying our predetermined exclusion criteria, a total of 16,758 questionnaires were included in our analyses (**Figure 1**). In total, 4,315 persons (26%) reported gastrointestinal symptoms, with a median symptom duration of 8 years (interquartile range 3–18 years). Compared to participants not reporting symptoms, those with gastrointestinal symptoms were younger (48.9±16 vs. 50.2±16 years), more often female (66% vs. 53%), and reported more frequently use of any medication (overall 80% vs 67%; **Table 1**).

**Table 1:** Population characteristics and factors associated with gastrointestinal symptom presence.

	Respondents with gastrointestinal symptoms	Respondents without gastrointestinal symptoms	Unadjusted OR (95% CI)	Adjusted OR (95% CI)
	n/N	n/N		
Mean age (±SD)	48.9 (16)	50.2 (16)	–	–
≥65 years (%)	747/4,300 (17)	2,451/12,389 (20)	0.85 (0.78–0.93)	0.75 (0.65–0.87)
Female (%)	2,784/4,217 (66)	6,448/12,079 (53)	1.70 (1.58–1.83)	1.59 (1.43–1.77)
Body mass index ≥25 kg/m^2^ (%)	2,067/4,224 (49)	5,549/12,213 (45)	1.15 (1.07–1.23)	1.02 (0.92–1.13)
Smoking (%)	840/4,247 (20)	2,089/12,242 (17)	1.20 (1.10–1.31)	1.12 (0.98–1.27)
Excessive alcohol consumption* (%)	394/2,919 (14)	1,229/9,266 (13)	1.02 (0.90–1.15)	0.93 (0.81–1.07)
Excessive coffee consumption† (%)	352/3,612 (10)	1,241/10,828 (12)	0.83 (0.74–0.95)	0.87 (0.74–1.02)
Co-morbidity (%)				
Diabetes mellitus	238/4,315 (6)	639/12,443 (5)	1.08 (0.93–1.26)	0.85 (0.64–1.13)
Rheumatoid arthritis	316/4,315 (7)	457/12,443 (4)	2.07 (1.79–2.40)	1.27 (0.99–1.62)
Asthma/COPD	393/4,315 (9)	651/12,443 (5)	1.82 (1.59–2.07)	1.47 (1.21–1.79)
Medication use				
PPIs§	1,161/4,315 (27)	610/12,443 (5)	7.14 (6.43–7.94)	9.28 (7.91–10.9)
H2RAs**	212/4,315 (5)	75/12,443 (1)	8.52 (6.53–11.1)	9.93 (6.72–14.7)
Antacids	588/4,315 (14)	437/12,443 (4)	4.33 (3.81–4.93)	4.22 (3.53–5.05)
Paracetamol	2,529/4,315 (59)	5,763/12,443 (46)	1.64 (1.53–1.76)	1.33 (1.20–1.47)
NSAIDs¶	1,076/4,315 (25)	2,157/12,443 (17)	1.58 (1.46–1.72)	1.02 (0.90–1.15)
Antiplatelet therapy#	462/4,315 (11)	1,221/12,443 (10)	1.10 (0.98–1.23)	1.08 (0.89–1.30)
Antidepressants	278/4,315 (6)	423/12,443 (3)	1.96 (1.68–2.29)	1.56 (1.22–2.00)
Statins	435/4,315 (10)	1,285/12,443 (10)	0.97 (0.87–1.09)	0.75 (0.61–0.93)
Oral contraceptives	339/4,315 (8)	716/12,443 (6)	1.40 (1.22–1.60)	1.22 (0.99–1.51)
Beta blockers	487/4,315 (11)	1,286/12,443 10)	1.10 (0.99–1.23)	0.87 (0.71–1.06)
ACE-inhibitors	227/4,315 (5)	727/12,443 (6)	0.90 (0.77–1.04)	0.81 (0.62–1.06)
Angiotensin-receptor antagonist	186/4,315 (4)	517/12,443 (4)	1.04 (0.88–1.23)	0.77 (0.58–1.03)
Diuretics	390/4,315 (9)	1,052/12,443 (9)	1.08 (0.95–1.22)	0.85 (0.68–1.06)

N/D = not determined.

* Excessive alcohol consumption is 14 units or more a week for women and 21 units or more a week for men.

† Excessive coffee consumption was defined as 42 cups a week and more.

§ PPI: proton pump inhibitor.

** H2RA: histamine-2 receptor antagonist.

¶ NSAIDs: non-steroidal anti-inflammatory drugs.

# Antiplatelet therapy: low-dose acetylsalicylic acid, carbasalate calcium, clopidogrel and dipyridamol are taken together.

The most frequently reported upper gastrointestinal symptoms were bloating (63%) and belching (45%; **Table 2**). Flatulence (71%) and borborygmi (60%) were the most frequently reported lower gastrointestinal tract symptoms (**Table 3**). Distribution of symptom presence among different age categories is depicted in **Table 4**.

**Table 2:** Type and frequency of upper gastrointestinal symptoms in respondents experiencing gastrointestinal symptoms.

	Number of respondents, n/N (%)
**Epigastric pain**	
** In general**	1,407/3,842 (36.6)
** During daytime**	1,422/3,565 (39.9)
** At night**	867/3,452 (25.1)
**Heartburn**	
** In general**	1,366/3,916 (34.9)
** During daytime**	1,250/3,537 (35.3)
** At night**	953/3,494 (27.3)
**Regurgitation**	1,545/4,012 (38.5)
**Belching**	1,884/4,166 (45.2)
**Empty feeling**	1,008/4,039 (25.0)
**Bloating**	2,627/4,164 (63.1)
**Nausea**	1,278/4,139 (30.9)
**Vomiting**	327/4,109 (8.0)
**Loss of appetite**	796/4,119 (19.3)
**Early satiety**	1,409/4,121 (34.2)
**Haematemesis**	36/4,091 (0.9)
**Dysphagia**	
** For liquids**	141/4,088 (3.4)
** For solid food**	257/3,957 (6.5)

**Table 3:** Type and frequency of lower gastrointestinal symptoms in respondents experiencing gastrointestinal symptoms.

	Number of respondents,n/N (%)
**Lower abdominal pain**	
** In general**	1,755/4,075 (43.1)
** Postprandial**	1,176/3,477 (33.8)
** Pre-prandial**	726/3,379 (21.5)
** No reduction after defecation**	867/3,338 (26.0)
**Flatulence**	2,965/4,193 (70.7)
**Borborygmi**	2,479/4,138 (59.9)
**Abnormal defecation**	
** Black stools**	338/3,714 (9.1)
** Blood**	187/3,708 (5.0)
** Mucous**	557/3,751 (14.8)
** Frequently hard**	1,418/3,810 (37.2)
** Diarrhoea**	1,330/3,812 (34.9)
** Constipation**	1,060/3,859 (27.5)
** Alternately solid or loose**	1,939/3,979 (48.7)
** Frequently painful**	908/3,818 (23.8)
** Strong urgency**	1,541/3,853 (40.0)
** Incomplete**	1,143/3,811 (30.0)
** Fatty stools**	1,012/3,882 (26.1)

**Table 4:** Symptom presence in respondents with gastrointestinal symptoms at various age categories.

	Age categories					
	18–30 years	31–40 years	41–50 years	51–60 years	61–70 years	≥71 years
	n/N (%)	n/N (%)	n/N (%)	n/N (%)	n/N (%)	n/N (%)
**Epigastric pain in general**	257/650 (39.5)	238/627 (38.0)	303/799 (37.9)	323/856 (37.7)	196/594 (33.0)	88/305 (28.9)
**Belching**	335/672 (49.9)	288/651 (44.2)	396/870 (45.5)	428/932 (45.9)	278/678 (41.0)	153/349 (43.8)
**Bloating**	486/667 (72.9)	441/647 (68.2)	579/865 (66.9)	583/942 (61.9)	351/678 (51.8)	181/352 (51.4)
**Heartburn in general**	184/666 (27.6)	223/636 (35.1)	321/822 (39.1)	324/853 (38.0)	200/614 (32.6)	108/314 (34.4)
**Borborygmi**	511/669 (76.4)	411/644 (63.8)	523/864 (60.5)	518/930 (55.7)	331/669 (49.5)	179/350 (51.1)
**Constipation**	237/654 (36.2)	194/618 (31.4)	224/808 (27.7)	203/851 (23.9)	111/604 (18.4)	86/311 (27.7)

The overall prevalence of gastrointestinal symptoms decreased with ageing. This was apparent in females (P<0.01), but not in males (P = 0.22; **Table S1)**. There was no effect of Body Mass Index on upper versus lower gastrointestinal symptoms (data not shown).

### Factors Associated with Gastrointestinal Symptoms

We found that respondents with gastrointestinal symptoms more frequently reported acid suppressive medication use. Other factors that were also more frequently reported in respondents with gastrointestinal symptoms are depicted in **Table 1**.

After adjustment, female gender (adjusted OR (aOR) 1.59, 95% CI 1.43–1.77), asthma/COPD (aOR 1.47, 95% CI 1.21–1.79), use of paracetamol (aOR 1.33, 95% CI 1.20–1.47), antidepressants (aOR 1.56, 95% CI 1.22–2.00) and use of acid-suppressive medication (antacids aOR 4.22, 95% CI 3.53–5.05, H2RAs aOR 9.93, 95% CI 6.72–14.7, PPIs aOR 9.29, 95% CI 7.91–10.9) remained independently associated with a higher risk for presence of gastrointestinal symptoms (**Table 1**). Age ≥65 years (aOR 0.75, 95% CI 0.65–0.87), and use of statins (aOR 0.75, 95% CI 0.61–0.93) were independently associated with a lower risk for presence of gastrointestinal symptoms. In the univariable analysis obesity was associated with presence of gastrointestinal symptoms (OR 1.15, 95% CI 1.10–1.31), but this association was lost after adjustment (aOR 1.02, 95% CI 0.92–1.13).

### Health-related Quality of Life

The mean utility for health-related quality of life was statistically significantly lower for respondents with gastrointestinal symptoms (0.81, SD 0.21) compared to respondents without gastrointestinal symptoms (0.92, SD 0.14, p<0.01). This difference was statistically significant (p<0.01) for all dimensions, and most pronounced for dimensions “pain/discomfort”, “anxiety/depression”, and “usual activities” (**Table 5**). The gastrointestinal symptom score (VAS) correlated negatively with health-related quality of life (EQ VAS) with a Spearman correlation of −0.57 (p<0.01), indicating that persons with more severe gastrointestinal symptoms reported a lower health-related quality of life. The following individual symptoms were associated with the lowest health-related quality of life: haematemesis (0.54, SD 0.32), dysphagia for liquid (0.59, SD 0.32) and solid intake (0.62, SD 0.30) and vomiting (0.66, SD 0.30; **Table S2**).

**Table 5:** Health-related quality of life in respondents with and without gastrointestinal symptoms.

	Respondents with gastrointestinalsymptoms	Respondents without gastrointestinalsymptoms	
EQ-5D dimension	n/N (%)	n/N (%)	P-value
**Mobility**			<0.001
No problems	3,440/4,203 (81.8)	10,880/12,194 (89.2)	
Some problems	745/4,203 (17.7)	1,290/12,194 (10.6)	
Extreme problems	18/4,203 (0.4)	24/12,194 (0.2)	
**Self-care**			<0.001
No problems	4,039/4,191 (96.4)	11,904/12,154 (97.9)	
Some problems	134/4,191 (3.2)	219/12,154 (1.8)	
Extreme problems	18/4,191 (0.4)	31/12,154 (0.3)	
**Usual activities**			<0.001
No problems	3,179/4,213 (75.5)	11,029/12,173 (90.6)	
Some problems	952/4,213 (22.6)	1,065/12,173 (8.7)	
Extreme problems	82//4,213 (1.9)	79/12,173 (0.6)	
**Pain/discomfort**			<0.001
No problems	1,715/4,209 (40.7)	9,424/12,128 (77.7)	
Some problems	2,263/4,209 (53.8)	2,559/12,128 (21.1)	
Extreme problems	231/4,209 (5.5)	145/12,128 (1.2)	
**Anxiety/depression**			<0.001
No problems	2,893/4,215 (68.6)	10,559/12,131 (87.0)	
Some problems	1,185/4,215 (28.1)	1,485/12,131 (12.2)	
Extreme problems	137/4,215 (3.3)	87/12,131 (0.7)	

## Discussion

We found that 26% of the general population reported gastrointestinal symptoms, with a median duration of eight years. Our study identifies female gender, asthma/COPD, use of paracetamol, antidepressants and acid suppressive medication use as risk factors that were independently associated with a higher prevalence of gastrointestinal symptoms. Older age and statin use protected against gastrointestinal symptoms. Respondents with gastrointestinal symptoms had an impaired health-related quality of life. In comparison to other, older, studies in the field [1], [3]–[12], we found a similar prevalence of gastrointestinal symptoms in the community. This suggests that the effect of time is limited, although there are a plethora of differences between our study and others, most importantly the definitions used to assess prevalence of gastrointestinal symptoms.

We found that females more frequently reported gastrointestinal symptoms, which is in line with other studies regarding gastrointestinal symptoms [9], [25], [33]. In our study, presence of asthma or COPD was an independent risk factor. Presence of asthma is associated with GERD, [34], [35], and recent studies indicate an increased prevalence of GERD symptoms in patients with COPD [36]–[38]. Medication use, and especially paracetamol, antidepressants, and acid-suppressive medication contributed significantly to presence of gastrointestinal symptoms in our large population-based survey. The independent association found for paracetamol, probably stems from the use of paracetamol as a panacea for gastrointestinal symptoms. We surmise that this hypothesis also applies to the relation between acid-suppressive medication and gastrointestinal symptoms. The association between antidepressants and gastrointestinal symptoms is complex due to the interactions between: 1) depression and gastrointestinal symptoms; 2) depression and antidepressant use, and 3) antidepressant use and gastrointestinal symptoms [39]–[48].

A total of 11% of our studied population reported PPI use, 2% H2RA use and 6% antacid use. This is much lower than the use of so-called ‘indigestion remedies’ in a study by Jones *et al*. prior to the PPI era, in which 47–55% of respondents reported any use of this medication class [3]. Use of PPIs was strongly associated with gastrointestinal symptom presence in our study (adjusted OR 9.28, 95% CI 7.91–10.9). This can by explained by a combination of indication bias, partial responsiveness on PPI therapy and assessment of both upper and lower gastrointestinal symptom presence. However, the prevalence of gastrointestinal symptoms would be even higher if we would include respondents with acid suppressive medication without current gastrointestinal symptoms in our prevalence.

In a recently published study, the prevalence of *upper* gastrointestinal symptoms in The Netherlands, the country where our study was performed, was 24% [21]. We found an almost similar prevalence (26%), but we assessed both upper *and* lower gastrointestinal symptoms. We found that the majority of respondents with gastrointestinal symptoms had both upper and lower gastrointestinal symptoms. Respondents with upper gastrointestinal symptoms have a higher risk for associated lower gastrointestinal symptoms. This is in line with a Japanese study that reported overlap of GERD, functional dyspepsia and IBS in 45% of their studied population [23]. Moreover, in the natural history of functional gastrointestinal disorders many patients frequently switch between upper and lower gastrointestinal symptoms [49].

We also reported the impact of gastrointestinal symptoms on health-related quality of life. Gastrointestinal symptoms were associated with a disutility of 0.11, which was in line with a disutility for dyspeptic symptoms of 0.09 in another study [50]. Furthermore, we observed that more severe symptoms correlated with a lower health-related quality of life (**Table S2**). By presenting a wide variety of utilities, we have delivered input for cost-utility studies. These studies become more and more important, and are incorporated in clinical guidelines, e.g. by the National Institute for Health and Care and Excellence (NICE).

The major strength of our study is that we examined commonly experienced symptoms in the community by use of a broad definition. Secondly, in order to attain a representative sample, persons were randomly selected via databases of local authorities without stringent in- and exclusion criteria. Third, we studied gastrointestinal symptoms overall and per symptom instead of in clusters of gastrointestinal symptoms.

Our study design comes with limitations. We cannot exclude response bias, as our response rate was 35%. Response rates in epidemiological studies are declining the last decades and this problem is faced by multiple researchers [51], [52]. A study by Galea et al. describes that a low response rate is not inevitably leading to substantial changes in outcomes [51]. Since our study was performed with postal questionnaires, in comparison to digital surveys, our response rate is actually not that low and still within the range of an acceptable response rate according to Galea et al. Due to concealment we were not able to perform non-responder research. We tried to minimize this bias by inviting all subjects with a personalized cover letter, and we asked explicitly to return the questionnaire, irrespective of presence of gastrointestinal symptoms. Seventy-four percent of all responders did not report the presence of gastrointestinal symptoms. Furthermore, the prevalence of co-morbidities in our study cohort resembles the prevalence in the general population [53]. Therefore, we assume that response bias might be limited. We also cannot exclude that bias was introduced by exclusion of individuals with incomplete information about gastrointestinal symptoms.

The results of our study will refresh awareness among healthcare providers on the high prevalence of gastrointestinal symptoms in the general population. Future research should focus on new targets to effectively treat patients with gastrointestinal symptoms, as we have shown that even many users of acid suppressive medication still report presence of symptoms.

In conclusion, despite increased treatment options and alterations in risk factors, the prevalence of gastrointestinal symptoms in the western community remains high and is associated with a considerable decrease in health-related quality of life.

## Supporting Information

Table S1 **Prevalence of gastrointestinal symptoms per age category and gender.**
(DOC)

Table S2 **Impact of individual gastrointestinal symptoms on health-related quality of life.**
(DOC)
