# Supplementary material for: Gastrointestinal Symptoms are Still Prevalent and Negatively Impact Health-Related Quality of Life: A Large Cross-Sectional Population Based Study in The Netherlands
Source: PLoS One. 2013 Jul 29;8(7):e69876. doi: 10.1371/journal.pone.0069876 (PMC3726702; doi:10.1371/journal.pone.0069876)
Supplement: Table S1 — Prevalence of gastrointestinal symptoms per age category and gender. (DOC) [file pone.0069876.s001.doc]

**Table S1: Prevalence of gastrointestinal symptoms per age category and gender**

|  | **Age categories (in years)** | | | | | |
| --- | --- | --- | --- | --- | --- | --- |
|  | **18 - 30**  **n / N (%)** | **31 - 40**  **n / N (%)** | **41 - 50**  **n / N (%)** | **51 - 60**  **n/ N (%)** | **61 - 70**  **n / N (%)** | **≥ 71**  **n / N (%)** |
| **Male gender** | 161 / 764 (21.1) | 201 / 996 (20.2) | 290 / 1,404 (20.7) | 360 / 1,712 (21.0) | 274 / 1,408 (19.5) | 143 / 756 (18.9) |
| **Female gender** | 505 / 1,513 (33.4) | 448 / 1,512 (29.6) | 576 / 1,886 (30.5) | 581 / 1,932 (30.1) | 432 / 1,493 (28.9) | 233 / 862 (27.0) |
