# Supplementary material for: Gastrointestinal Symptoms are Still Prevalent and Negatively Impact Health-Related Quality of Life: A Large Cross-Sectional Population Based Study in The Netherlands
Source: PLoS One. 2013 Jul 29;8(7):e69876. doi: 10.1371/journal.pone.0069876 (PMC3726702; doi:10.1371/journal.pone.0069876)
Supplement: Table S2 — Impact of individual gastrointestinal symptoms on health-related quality of life. (DOC) [file pone.0069876.s002.doc]

**Table S2: Impact of individual gastrointestinal symptoms on health-related quality of life**

| **Symptom** | | **Dutch Utility score**  **Mean (±SD)** |
| --- | --- | --- |
| Epigastric pain | |  |
|  | In general | 0.74 (0.24) |
|  | During daytime | 0.81 (0.19) |
|  | At night | 0.72 (0.26) |
| Heartburn | |  |
|  | In general | 0.78 (0.23) |
|  | During daytime | 0.78 (0.23) |
|  | At night | 0.77 (0.24) |
| Regurgitation | | 0.77 (0.23) |
| Belching | | 0.78 (0.22) |
| Empty feeling | | 0.75 (0.24) |
| Bloating | | 0.78 (0.22) |
| Nausea | | 0.71 (0.26) |
| Vomiting | | 0.66 (0.30) |
| Loss of appetite | | 0.67 (0.27) |
| Early satiety | | 0.73 (0.25) |
| Haematemesis | | 0.53 (0.32) |
| Dysphagia | |  |
|  | Liquid | 0.59 (0.32) |
|  | Solid | 0.62 (0.30) |
| Lower abdominal pain | |  |
|  | In general | 0.74 (0.24) |
|  | Postprandial | 0.75 (0.24) |
|  | Pre-prandial | 0.71 (0.26) |
|  | No reduction after defecation | 0.73 (0.25) |
| Flatulence | | 0.79 (0.21) |
| Borborygmi | | 0.78 (0.22) |
| Abnormal defecation | |  |
|  | Black stools | 0.71 (0.27) |
|  | Blood | 0.73 (0.26) |
|  | Mucous | 0.73 (0.25) |
|  | Frequently hard | 0.76 (0.23) |
|  | Diarrhoea | 0.76 (0.23) |
|  | Constipation | 0.73 (0.25) |
|  | Alternately solid or loose | 0.78 (0.22) |
|  | Frequently painful | 0.70 (0.26) |
|  | Strong urgency | 0.76 (0.23) |
|  | Incomplete | 0.74 (0.24) |
|  | Fatty stools | 0.74 (0.24) |
